# Cerebral Blood Volume During Neonatal Transition in Term and Preterm Infants With and Without Respiratory Support

**DOI:** 10.3389/fped.2018.00132

**Published:** 2018-05-04

**Authors:** Bernhard Schwaberger, Gerhard Pichler, Corinna Binder-Heschl, Nariae Baik-Schneditz, Alexander Avian, Berndt Urlesberger

**Affiliations:** ^1^Research Unit for Cerebral Development and Oximetry, Medical University of Graz, Graz, Austria; ^2^Division of Neonatology, Department of Pediatrics, Medical University of Graz, Graz, Austria; ^3^Institute for Medical Informatics, Statistics and Documentation, Medical University of Graz, Graz, Austria

**Keywords:** cerebral blood volume, near-infrared spectroscopy (NIRS), neonatal transition, ventilation induced brain injury, preterm infants, ventilation

## Abstract

**Background:** Recently, we demonstrated that in healthy newborn infants cerebral blood volume (CBV) was decreasing continuously after birth. We hypothesized that this was due to the increase in oxygen delivery to the brain during neonatal transition. Thus delayed cerebral oxygen delivery in infants in need for respiratory support (RS) during postnatal stabilization might influence changes in CBV.

**Objective:** Aim of the study was to evaluate transitional changes in CBV immediately after birth in term and preterm infants with and without need of RS.

**Methods:** We performed a *post-hoc* analysis of data collected as primary and secondary outcome parameters in prospective observational studies and randomized controlled trials at the Medical University of Graz (Austria). NIRS measurements by using “NIRO 200-NX” (Hamamatsu, Japan) were carried out over the first 15 min after birth in term and preterm infants delivered by cesarean section with and without requirement for RS.

**Results:** In 204 neonates, we observed a significant decrease in CBV within the first 15 min after birth (*p* < 0.001) with a trend toward smaller ΔCBV in neonates receiving RS (*p* = 0.097) compared to neonates without RS. Differences of ΔCBV between groups reached statistically significance (*p* < 0.05) at minutes 2, 6, and 7, and showed a trend (*p* < 0.1) at minutes 3, 4, and 5. After adjusting for gestational age, these differences became smaller and failed to reach significance.

**Conclusions:** We observed a significant decrease of CBV in term and preterm infants with and without RS. Interestingly, ΔCBV was smaller in the first 7 min in neonates with RS reaching statistically significance (*p* < 0.05) at minutes 2, 6, and 7. This study cannot differentiate, whether RS itself or the condition leading to requirement for RS is responsible for the observed CBV behavior.

## Introduction

Hemodynamic disturbances have been shown to potentially result in alterations of cerebral perfusion and subsequent brain injury in newborn infants (1–3). This might be of particular relevance for preterm infants in which cerebral autoregulation may not be fully present immediately after birth (2–4).

The use of near-infrared spectroscopy (NIRS) during immediate neonatal transition enables a non-invasive method to evaluate cerebral hemodynamics. Most of the previously published NIRS studies in newborns during neonatal transition reported exclusively on cerebral tissue oxygenation (1, 5–15). However, since NIRS technology further provides measurements of changes in total hemoglobin (ΔHbT), the evaluation of changes in cerebral blood volume (ΔCBV) is feasible. Recently, our research group demonstrated that in healthy newborn infants CBV is continuously decreasing during the first 15 min after birth, hypothesizing that this was mainly due to the increase in oxygen delivery to the brain resulting in cerebral vasoconstriction (16).

Newborn infants receiving respiratory support (RS) during postnatal stabilization show a delayed increase in oxygen delivery (12), which potentially influences transitional CBV changes. The occurrence of hemodynamic disturbances in ventilated infants is discussed to be one pathway resulting in ventilation-induced brain injury, especially in newborn infants with impaired mechanisms of autoregulation (4). It has been shown, that significantly reduced cerebral tissue oxygen saturation during neonatal transition was associated with severe intraventricular hemorrhage (1). Not only pO_2_ and pCO_2_ may influence CBV, but compromised cerebral perfusion might further be caused by an increased pulmonary resistance and decreased cardiac output due to over-distension of alveoli and compression of pulmonary capillaries (2, 3). This indicates that monitoring of cerebral oxygenation and perfusion might be beneficial in infants with RS during postnatal stabilization. The aim of the present study was to analyze the potential influence of RS on the course of CBV during the transition of the newborn. We hypothesized that the physiological CBV decrease during neonatal transition would be diminished in infants receiving RS showing smaller ΔCBV compared to those infants with normal transition.

## Materials and methods

### Study design

We performed a *post-hoc* analysis of data collected as primary and secondary outcome parameters in three prospective observational studies and one randomized controlled trial at the Medical University of Graz; Austria (5, 16–18). The Regional Committee on Biomedical Research Ethics approved all of the included studies. Between October 2010 and January 2015 term and preterm infants delivered by cesarean section with and without the need of RS were included after a written informed consent was obtained from the parents prior to birth. Due to technical reasons the NIRS measurements couldn't be performed in the delivery room next to the mother. Therefore, vaginally born infants were excluded to avoid a delay or disturbance of immediate skin-to-skin contact with the mother. Further exclusion criteria were presence of congenital malformations, inherited disorders of metabolism, decision to not provide full life support, and application of sustained lung inflations during postnatal stabilization.

### Procedure

After cord clamping, routinely performed after 30 s, infants were placed on the resuscitation table under an overhead heater. The newborn infants were dried and stimulated by using warm cotton diapers to induce effective breathing. In case of obvious or suspected upper airway obstruction immediate suction of the oropharynx was performed. If necessary, RS by using a “Neopuff Infant T-Piece Resuscitator” (Perivent, Fisher & Paykel Healthcare; New Zealand) was provided via face mask of appropriate size (LSR Silicon mask no. 0/0 or 0/1, Laerdal; Norway) according to the guidelines (19–21) either by applying PPV or CPAP depending on the breathing efforts of the patient. The pre-set FiO_2_ was 0.21 in term infants and 0.3 in preterm infants and was adapted to achieve defined oxygen saturation targets during immediate postnatal transition (22).

As soon as possible, scientific staff members attached the NIRS transducer on the newborn's right forehead by using gauze bandage without disturbing routine medical care. NIRS measurements were carried out with a “NIRO 200-NX” tissue oxygenation monitor (Hamamatsu; Japan). By using NIRS, cerebral tissue oxygenation index (cTOI) and changes in ΔHbT were measured with a sample rate of 2 Hertz. Additionally both, preductal arterial oxygen saturation (SpO_2_) and HR, were continuously monitored by using pulse-oximetry measured on the right wrist (M1193A Neonate Silicon Wrap, Philips; the Netherlands). The measurements were conducted over the first 15 min after birth. Singular measurements of blood pressure and rectal body temperature were recorded between minute 10 and 15 after birth and displayed on the “IntelliVue MP30/X2” monitor (Philips; the Netherlands).

For analysis all parameters and video recordings were stored using a multichannel system “alpha-trace digital MM” (BEST Medical Systems; Austria).

### Statistics

In each neonate, ΔHbT values for each minute after birth were calculated by subtracting the mean ΔHbT value at minute 15 from the mean ΔHbT value of the related minute. The 15 min value was used as reference value, because at that time point NIRS signal quality was most stable and reliable. Next, ΔHbT values were converted to ΔCBV by the following equation, whereby Hb represents the hemoglobin concentration (g/dl) (23):

(1) $$\Delta CBV\;=\Delta HbT*\frac{0.89}{Hb}$$

For the calculation either the actual Hb level of the individual (from routinely performed blood sampling within 30 min after birth) or the averaged group Hb of term or preterm infants (if the individual value was not available) was used.

Demographic variables are presented as absolute and relative counts, mean, and standard deviation (SD) or median and interquartile range (IQR), as appropriate. Comparisons of categorical baseline characteristics between infants with and without RS and between preterm and term infants were made using chi-square test, *t-*test or Mann–Whitney *U*-test, as appropriate. Data of ΔCBV, cTOI, SpO_2_, and HR are presented as mean and 95% confidence interval (95%CI). We investigated the courses of ΔCBV, cTOI, SpO_2_, and HR within the first 15 min after birth using a linear mixed model with fixed effects for time, RS (with RS vs. without RS), and gestational age (preterm vs. term). Since most of preterm neonates (82.2%) required RS and most of term neonates (88.1%) did not receive RS, we tested a model including RS without considering gestational age (model 1) and a model including RS and gestational age (model 2). The decision which of these models to be used was based on a REML-based likelihood ratio test. In all models a first order autoregressive covariance structure was used. The autoregressive covariance structure assumes a systematically decreasing correlation with increasing distance between time points. Therefore, adjacent time points will have the highest correlations. *Post-hoc* analyses for differences between groups for each minute were performed for the comparison of RS groups (with RS vs. without RS) and if model 2 was chosen also for gestational age groups (term infants vs. preterm infants). If model 1 (without comparison: term infants vs. preterm infants) was chosen, the model 2 is given in the supplement together with the model 1. A *p*-value < 0.05 was considered statistical significant. Statistical analyses were performed using SPSS Statistics 20.0.0 (IBM; USA).

## Results

In total, in 204 neonates measurements were performed during the study period including 45 preterm infants (37 with and 8 without RS) and 159 term infants (19 with and 140 without RS) born at a mean gestational age of 33+3 weeks (±15 days) and 38+6 weeks (±6 days), respectively. Thus, 56 newborn infants received RS during the transitional period, whereas 148 had a normal neonatal transition without the need of RS. As RS was according to individual needs of the infants, start points and duration of PPV and/or CPAP and extent of FiO_2_ were different in each subject. RS started at 2.5 (4.0) min, duration of RS within the first 15 min of life was 11.5 (9.0) min [median (interquartile range)].

Demographic and clinical data of newborn infants with and without RS during immediate postnatal transition are summarized in Table 1.

**Table 1:** Demographic and clinical characteristics of newborn infants with and without RS.

	**With RS (*n* = 56)**	**Without RS (*n* = 148)**	***p-*value**
Gestational age (wk), mean (±SD)	35.0 (3.2)	38.7 (1.2)	< 0.001
Birth weight (g), mean (±SD)	2,382 (916)	3,232 (502)	< 0.001
Head circumference (cm), mean (±SD)	32.2 (3.3)	34.6 (1.5)	< 0.001
Female sex, *n* (%)	31 (55)	76 (51)	0.609
Apgar at 1 min, median (IQR)	8 (8–8)	9 (9–9)	< 0.001
Apgar at 5 min, median (IQR)	9 (9–9)	10 (10–10)	< 0.001
Apgar at 10 min, median (IQR)	9 (9–10)	10 (10–10)	< 0.001
pH Umbilical artery, median (IQR)	7.29 (7.27–7.31)	7.29 (7.27–7.32)	0.695
Rectal body temperature (°C) at 15 min, mean (±SD)	36.7 (0.5)	36.7 (0.3)	0.794
Hemoglobin (g/dl) in umbilical cord blood, mean (±SD)	17.4 (3.1)	15.3 (1.6)	0.002
Systolic APB (mmHg) at 10 min, mean (±SD)	60.2 (9.4)	66.0 (9.4)	< 0.001
Mean APB (mmHg) at 10 min, mean (±SD)	41.2 (8.9)	45.4 (7.8)	0.003
Diastolic APB (mmHg) at 10 min, mean (±SD)	32.7 (9.4)	35.0 (10.3)	0.186

*ABP, arterial blood pressure; RS, respiratory support*.

Demographic and clinical data of preterm and term infants—irrespective of whether RS was applied or not—are summarized in Table 2.

**Table 2:** Demographic and clinical characteristics of preterm and term infants.

	**Preterm infants (*n* = 45)**	**Term infants (*n* = 159)**	***p*-value**
Gestational age (wk), mean (±SD)	33.5 (2.1)	38.9 (0.9)	< 0.001
Birth weight (g), mean (±SD)	1,900 (1,442–2580)	3,260 (2,954–3,480)	< 0.001
Head circumference (cm), median (IQR)	30.5 (29.0–38.0)	35.0 (34.0–35.5)	< 0.001
Female sex, *n* (%)	26 (58)	81 (51)	0.418
Apgar at 1 min, median (IQR)	8 (8–8)	9 (9–9)	< 0.001
Apgar at 5 min, median (IQR)	9 (8–9)	10 (10–10)	< 0.001
Apgar at 10 min, median (IQR)	9 (9–10)	10 (10–10)	< 0.001
pH Umbilical artery, mean (±SD)	7.30 (0.04)	7.28 (0.05)	0.015
Rectal body temperature (°C) at 15 min, mean (±SD)	36.7 (0.6)	36.7 (0.3)	0.546
Hemoglobin (g/dl) in umbilical cord blood, mean (±SD)	18.0 (3.0)	15.2 (1.6)	< 0.001
Systolic APB (mmHg) at 10 min, mean (±SD)	59.1 (10.0)	65.8 (9.2)	< 0.001
Mean APB (mmHg) at 10 min, mean (±SD)	40 (35–47)	45 (40–50)	0.002
Diastolic APB (mmHg) at 10 min, median (IQR)	32 (27–38)	35 (28–42)	0.090

*ABP, arterial blood pressure*.

### Cerebral blood volume

**Figure 1:**
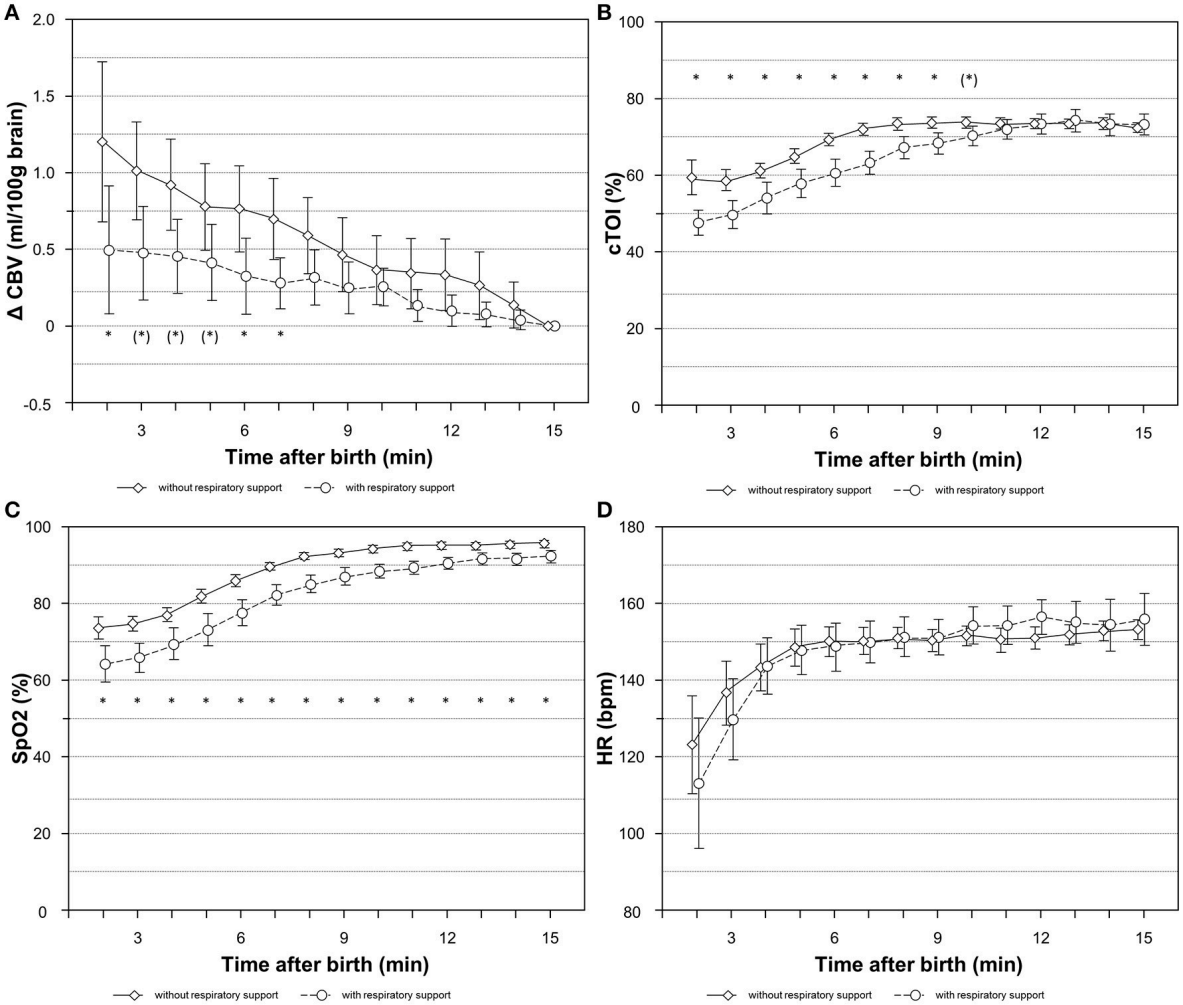
Courses of ΔCBV **(A)**, cTOI **(B)**, SpO_2_ **(C)**, and HR **(D)** during the first 15 min after birth in newborn infants with and without respiratory support. Values are mean (95%CI); ^*^p < 0.05, (*)*p* < 0.1, significances are calculated for group comparisons for each minute. bpm, beats per minute; CBV, cerebral blood volume; cTOI, cerebral tissue oxygenation index; HR, heart rate; SpO_2_, arterial oxygen saturation.

In the whole study population a significant decrease in CBV was observed within the first 15 min after birth (*p* < 0.001). Furthermore we observed a trend toward smaller ΔCBV in neonates with RS (*p* = 0.097), while the courses of ΔCBV were comparable between groups (*p* = 0.655). Differences of ΔCBV between groups reached statistically significance (*p* < 0.05) at minutes 2, 6, and 7. The ΔCBV values at minutes 3, 4, and 5 showed a trend (*p* < 0.10) toward a difference between groups (Figure 1A). The inclusion of the gestational age did not result in a significant better model fit (*p* = 0.172), meaning that gestational age could not explain further differences in ΔCBV between neonates. Nevertheless, gestational age was included in a sensitivity analysis, which resulted in comparable effects (Supplementary Tables 1, 2).

### cTOI, SpO_2_, and HR

In the whole study population cTOI (*p* < 0.001) and SpO_2_ values (*p* < 0.001) showed a significant increase during immediate postnatal transition. We found significant differences in cTOI and SpO_2_ levels between newborn infants with and without RS after birth showing significantly lower values in those infants who received RS (cTOI: *p* = 0.023; SpO_2_: *p* < 0.001). By comparing both groups, cTOI levels did not equalize before the 9th minute of age, whereas the differences of SpO_2_ continued until the end of the observational period of 15 min after birth (Figures 1B,C). cTOI and SpO_2_ values did not show significant differences comparing term to preterm infants (cTOI: *p* = 0.577; SpO_2_: *p* = 0.595). While courses of cTOI were comparable between term and preterm infants (cTOI: *p* = 0.379) SpO_2_ showed significant different courses between term and preterm infants (SpO_2_: *p* = 0.031) with a slightly steeper increase in term infants at the end of the observational period (Supplementary Table 2).

HR increased significantly (*p* < 0.001) in the study population. This significant increase was mainly due to an increase within the first 5 min after birth. HR values were higher in newborn infants with RS compared to newborn infants without RS (*p* = 0.007) (Figure 1D) and were higher in term compared to preterm infants (*p* = 0.003). Courses of HR were comparable between term and preterm infants (*p* = 0.218) (Supplementary Table 2).

## Discussion

To the best of our knowledge this is the first study that incorporates detailed analysis of CBV in term and preterm infants with and without the need of RS during immediate postnatal transition. We observed a significant decrease in CBV within the first 15 min after birth in both groups, but the ΔCBV was smaller in the first 7 min in neonates with RS. Whether RS itself or the condition of the infant leading to requirement for RS is responsible for the observed CBV behavior cannot be explained in detail with the present analysis.

Our findings are in accordance with recently published data by our study group demonstrating a significant decrease in CBV in healthy term infants after birth (16). We hypothesized that the transitional CBV decrease reflected a physiological response to changing blood gases within the autoregulatory capacity of cerebral vessels as pO_2_ increases and pCO_2_ decreases postnatally (16). Blood gas changes may occur to a different extent in different individuals during postnatal immediate transition, but generally during neonatal transition the changes in pO_2_ are more distinct, compared to changes in pCO_2_. Furthermore, our study group demonstrated that preterm infants receiving RS during neonatal transition showed a significantly lower cerebral tissue oxygenation compared to infants without requirement of RS (6, 12). Thus, decreased cerebral oxygenation in infants receiving RS potentially may be accompanied by cerebral vasodilatation to increase cerebral blood flow and improve oxygen delivery (24). The extent of CBV decrease due to cerebral vasoconstriction might substantially depend on pO_2_ levels, which increase less rapidly in infants requiring RS immediately after birth (6, 12, 25).

Moreover, we recently published a randomized pilot study showing an influence of sustained lung inflation on CBV behavior in preterm infants. Whereas in preterm infants receiving RS standard care (PPV or CPAP) after birth CBV was decreasing, CBV basically remained unchanged in preterm infants receiving one to three sustained lung inflations followed by PPV or CPAP during immediate transition (17). Based on these observation we hypothesized that RS itself might influence postnatal CBV changes, most probably by increasing the intrathoracic pressure leading to cerebral venous stasis and an impairment of venous return to the heart (17). The significant differences in the postnatal CBV behavior justify the exclusion of infants receiving sustained lung inflations from the present *post-hoc* analysis.

However, it has been shown that initial RS immediately after birth is a considerable risk factor for cerebral injury and local brain inflammation in newborn neonates, particularly in preterm infants who already are at a high risk due to brain immaturity (3). Even though there is growing evidence that ventilation-induced injuries occur as early as ventilation is initiated in the delivery room, PPV immediately after birth still is one of the least controlled interventions in preterm infant (3). By using a respiratory function monitor, potentially harmful high tidal volumes were demonstrated during postnatal stabilization in more than 85% of preterm infants receiving PPV via face mask (26). These high tidal volumes might influence cerebral perfusion and might have hazardous effects on the brain (2, 27). The present study cannot add to that topic, as the number of infants was too small to draw a conclusion in regard to cerebral injury. Therefore, further research is needed to optimize initial ventilatory strategies to potentially achieve improved cerebral outcome in newborn infants.

### Limitations

First, all the included infants were delivered by cesarean section and cord clamping was routinely performed 30 s after birth. Therefore it remains unclear, whether vaginal birth or delayed cord clamping would have resulted in a different CBV behavior.

NIRS assessment of the frontal cortical region by using a single transducer covers only a small part of the brain in supine positioned infants, although less likely, we cannot rule out that dynamics of CBV during transition might be different in different regions of the brain.

Furthermore, the conversion of NIRS derived ΔHbT to ΔCBV requires the individual Hb levels of every single patient. Unfortunately, it wasn't possible to obtain Hb levels in each patient, because the Regional Committee on Biomedical Research Ethics did not authorize blood sampling exclusively for study purposes. Therefore, in absence of the individual levels we used mean Hb values of the respective study group for calculating ΔCBV. Moreover, we cannot provide continuous pO_2_ or pCO_2_ values, which might have been beneficial for the interpretation of the CBV results.

Next, since groups differed in their gestational age, the influence of the gestational age on the primary could not be sufficient investigated. The influence of gestational age on ΔCBV should be a topic of further research.

Last, our results derived by *post-hoc* analysis need to be interpreted with caution and confirmation by other appropriately designed prospective studies is required.

## Conclusion

We observed a significant decrease of CBV in both groups, in infants undergoing normal transition and in infants in need for RS (either PPV or CPAP). Interestingly, ΔCBV was smaller in the first 7 min in neonates with RS. This study did not yet determine, whether RS itself or the condition of the infant leading to requirement for RS is responsible for the observed differences in CBV compared to healthy newborn infants, but we would hypothesize, that differences in cerebral oxygen delivery may explain this best. Our results are of particularly great interest, since hemodynamic disturbances resulting in changes in cerebral perfusion are discussed to be an important pathway to ventilator-induced brain injury occurring as early as ventilation is initiated in the delivery room.

## Author contributions

BS, GP, CB-H, NB-S, and BU: substantial contributions to the conception or design of the work; BS, GP, AA, and BU: the acquisition, analysis, or interpretation of data for the work; BS, GP, CB-H, NB-S, AA, and BU: drafting the work or revising it critically for important intellectual Content; BS, GP, CB-H, NB-S, AA, and BU: final approval of the version to be published; BS, GP, CB-H, NB-S, AA, and BU: agreement to be accountable for all aspects of the work in ensuring that questions related to the accuracy or integrity of any part of the work are appropriately investigated and resolved.

### Conflict of interest statement

The authors declare that the research was conducted in the absence of any commercial or financial relationships that could be construed as a potential conflict of interest.

## Abbreviations

ABP: arterial blood pressure
CBV: cerebral blood volume
CPAP: continuous positive airway pressure
cTOI: cerebral tissue oxygenation index
Hb: hemoglobin
ΔHbT: changes in the concentration of total hemoglobin
HR: heart rate
NIRS: near-infrared spectroscopy
PPV: positive pressure ventilation
RS: respiratory support
SpO_2_: arterial oxygen saturation.
