# Supplementary material for: Cerebral Blood Volume During Neonatal Transition in Term and Preterm Infants With and Without Respiratory Support
Source: Front Pediatr. 2018 May 4;6:132. doi: 10.3389/fped.2018.00132 (PMC5945863; doi:10.3389/fped.2018.00132)
Supplement: Supplementary file 1 [file Data_Sheet_1.docx]

Supplemental Table 1: Comparison of model 1 (without considering gestational age) and model 2 (with gestational age).

|  | **Model 1** | **Model 2** |
| --- | --- | --- |
| **Time** | <.001 | <.001 |
| **Group (with respiratory support vs. without respiratory support)** | .097 | .306 |
| **Time * group** | .655 | .656 |
| **Term vs. preterm neonates** |  | .694 |
| **Post Hoc Analysis:**  **with respiratory support vs. without respiratory support** | | |
| **Minute 2** | .034 | .113 |
| **Minute 3** | .055 | .170 |
| **Minute 4** | .089 | .229 |
| **Minute 5** | .087 | .227 |
| **Minute 6** | .033 | .120 |
| **Minute 7** | .046 | .151 |
| **Minute 8** | .161 | .345 |
| **Minute 9** | .264 | .479 |
| **Minute 10** | .570 | .801 |
| **Minute 11** | .288 | .508 |
| **Minute 12** | .225 | .432 |
| **Minute 13** | .334 | .561 |
| **Minute 14** | .618 | .847 |
| **Minute 15** | 1.000 | .825 |

Supplemental Table 2: Estimated means and 95% confidence intervals

|  | CBV | | | cTOI | | | SpO_2_ | | | HR | | |
| --- | --- | --- | --- | --- | --- | --- | --- | --- | --- | --- | --- | --- |
|  | estimated  mean | 95% CI | | estimated  mean | 95% CI | | estimated  mean | 95% CI | | estimated  mean | 95% CI | |
|  |  | lower  bound | upper  bound |  | lower  bound | upper  bound |  | lower  bound | upper  bound |  | lower  bound | upper  bound |
| Overall | 0.397 | 0.243 | 0.551 | 66.9 | 65.4 | 68.4 | 85.0 | 84.0 | 86.0 | 145.8 | 143.1 | 148.6 |
| time (min) |  |  |  |  |  |  |  |  |  |  |  |  |
| 2 | 0.747 | 0.541 | 0.954 | 55.0 | 52.9 | 57.1 | 67.5 | 66.0 | 68.9 | 113.0 | 107.9 | 118.1 |
| 3 | 0.689 | 0.492 | 0.886 | 54.3 | 52.2 | 56.3 | 69.9 | 68.6 | 71.3 | 127.3 | 122.8 | 131.7 |
| 4 | 0.632 | 0.438 | 0.825 | 57.4 | 55.4 | 59.4 | 72.9 | 71.6 | 74.2 | 142.4 | 138.2 | 146.6 |
| 5 | 0.602 | 0.412 | 0.793 | 61.1 | 59.1 | 63.1 | 77.4 | 76.1 | 78.7 | 147.1 | 142.8 | 151.3 |
| 6 | 0.559 | 0.368 | 0.749 | 64.7 | 62.8 | 66.7 | 81.7 | 80.4 | 83.0 | 148.3 | 144.1 | 152.6 |
| 7 | 0.501 | 0.311 | 0.691 | 67.5 | 65.5 | 69.5 | 85.5 | 84.2 | 86.8 | 148.6 | 144.4 | 152.9 |
| 8 | 0.451 | 0.261 | 0.641 | 70.0 | 68.0 | 71.9 | 88.6 | 87.3 | 89.9 | 150.3 | 146.1 | 154.5 |
| 9 | 0.357 | 0.167 | 0.547 | 70.6 | 68.7 | 72.6 | 90.0 | 88.7 | 91.3 | 149.9 | 145.6 | 154.1 |
| 10 | 0.310 | 0.120 | 0.499 | 71.6 | 69.6 | 73.6 | 91.2 | 89.9 | 92.5 | 151.7 | 147.5 | 155.9 |
| 11 | 0.237 | 0.048 | 0.427 | 72.3 | 70.4 | 74.3 | 92.0 | 90.7 | 93.3 | 151.5 | 147.3 | 155.7 |
| 12 | 0.215 | 0.025 | 0.404 | 73.1 | 71.1 | 75.1 | 92.7 | 91.4 | 94.0 | 152.8 | 148.5 | 157.0 |
| 13 | 0.169 | -0.021 | 0.358 | 73.5 | 71.5 | 75.5 | 93.4 | 92.1 | 94.7 | 152.6 | 148.3 | 156.8 |
| 14 | 0.085 | -0.104 | 0.274 | 73.1 | 71.1 | 75.1 | 93.5 | 92.2 | 94.8 | 152.7 | 148.5 | 157.0 |
| 15 | 0.000 | -0.189 | 0.189 | 72.6 | 70.6 | 74.6 | 94.0 | 92.7 | 95.3 | 153.6 | 149.4 | 157.8 |
| RS |  |  |  |  |  |  |  |  |  |  |  |  |
| without RS | 0.527 | 0.366 | 0.688 | 68.6 | 66.4 | 70.8 | 88.0 | 86.6 | 89.5 | 143.8 | 139.7 | 147.9 |
| with RS | 0.267 | 0.004 | 0.529 | 65.3 | 62.8 | 67.7 | 82.0 | 80.4 | 83.6 | 147.9 | 143.4 | 152.3 |
| GA |  |  |  |  |  |  |  |  |  |  |  |  |
| term |  |  |  | 68.0 | 66.1 | 70.0 | 85.4 | 84.1 | 86.6 | 149.1 | 145.6 | 152.7 |
| preterm |  |  |  | 65.8 | 63.0 | 68.6 | 84.7 | 82.8 | 86.5 | 142.5 | 137.4 | 147.7 |
| Time * RS |  |  |  |  |  |  |  |  |  |  |  |  |
| min: 2 / without RS | 0.97 | 0.75 | 1.20 | 60.3 | 57.6 | 63.1 | 71.6 | 69.6 | 73.6 | 116.5 | 109.8 | 123.1 |
| min: 2 / with RS | 0.52 | 0.18 | 0.87 | 49.7 | 46.2 | 53.2 | 63.3 | 60.9 | 65.7 | 109.6 | 101.4 | 117.8 |
| min: 3 / without RS | 0.89 | 0.69 | 1.10 | 57.9 | 55.3 | 60.5 | 73.7 | 71.9 | 75.4 | 128.1 | 122.5 | 133.7 |
| min: 3 / with RS | 0.49 | 0.15 | 0.82 | 50.6 | 47.3 | 54.0 | 66.2 | 64.0 | 68.4 | 126.4 | 119.2 | 133.7 |
| min: 4 / without RS | 0.80 | 0.60 | 1.00 | 60.2 | 57.6 | 62.8 | 76.8 | 75.1 | 78.5 | 140.2 | 134.9 | 145.5 |
| min: 4 / with RS | 0.46 | 0.13 | 0.79 | 54.6 | 51.2 | 57.9 | 69.1 | 66.9 | 71.3 | 144.6 | 137.5 | 151.7 |
| min: 5 / without RS | 0.77 | 0.57 | 0.97 | 63.9 | 61.3 | 66.5 | 81.6 | 79.9 | 83.3 | 145.4 | 140.0 | 150.7 |
| min: 5 / with RS | 0.44 | 0.11 | 0.76 | 58.4 | 55.1 | 61.7 | 73.2 | 71.1 | 75.4 | 148.8 | 141.7 | 155.8 |
| min: 6 / without RS | 0.77 | 0.57 | 0.96 | 68.3 | 65.7 | 70.9 | 85.6 | 83.9 | 87.4 | 146.8 | 141.5 | 152.1 |
| min: 6 / with RS | 0.35 | 0.03 | 0.68 | 61.2 | 57.9 | 64.6 | 77.7 | 75.6 | 79.9 | 149.9 | 142.8 | 156.9 |
| min: 7 / without RS | 0.69 | 0.50 | 0.89 | 71.0 | 68.4 | 73.6 | 89.4 | 87.7 | 91.1 | 147.3 | 142.0 | 152.6 |
| min: 7 / with RS | 0.31 | -0.02 | 0.63 | 64.0 | 60.7 | 67.3 | 81.6 | 79.4 | 83.7 | 150.0 | 142.9 | 157.1 |
| min: 8 / without RS | 0.59 | 0.39 | 0.78 | 72.4 | 69.8 | 75.0 | 92.0 | 90.3 | 93.7 | 148.1 | 142.8 | 153.4 |
| min: 8 / with RS | 0.32 | -0.01 | 0.64 | 67.5 | 64.2 | 70.8 | 85.2 | 83.0 | 87.3 | 152.4 | 145.4 | 159.5 |
| min: 9 / without RS | 0.46 | 0.27 | 0.66 | 72.6 | 70.0 | 75.2 | 92.8 | 91.1 | 94.5 | 147.5 | 142.2 | 152.8 |
| min: 9 / with RS | 0.25 | -0.07 | 0.57 | 68.7 | 65.4 | 72.0 | 87.1 | 85.0 | 89.3 | 152.2 | 145.2 | 159.3 |
| min: 10 / without RS | 0.36 | 0.17 | 0.56 | 72.7 | 70.1 | 75.2 | 93.9 | 92.2 | 95.7 | 148.7 | 143.4 | 154.0 |
| min: 10 / with RS | 0.25 | -0.07 | 0.58 | 70.6 | 67.3 | 73.9 | 88.4 | 86.3 | 90.6 | 154.7 | 147.6 | 161.8 |
| min: 11 / without RS | 0.34 | 0.14 | 0.54 | 72.4 | 69.8 | 75.0 | 94.6 | 92.9 | 96.3 | 147.6 | 142.3 | 152.9 |
| min: 11 / with RS | 0.13 | -0.19 | 0.46 | 72.3 | 69.0 | 75.6 | 89.4 | 87.3 | 91.6 | 155.4 | 148.3 | 162.4 |
| min: 12 / without RS | 0.33 | 0.13 | 0.53 | 72.5 | 69.9 | 75.1 | 94.8 | 93.1 | 96.5 | 148.1 | 142.8 | 153.4 |
| min: 12 / with RS | 0.10 | -0.23 | 0.42 | 73.6 | 70.3 | 76.9 | 90.6 | 88.4 | 92.7 | 157.5 | 150.4 | 164.5 |
| min: 13 / without RS | 0.26 | 0.06 | 0.46 | 72.4 | 69.9 | 75.0 | 94.9 | 93.2 | 96.6 | 149.0 | 143.7 | 154.3 |
| min: 13 / with RS | 0.08 | -0.25 | 0.40 | 74.5 | 71.2 | 77.8 | 91.9 | 89.7 | 94.0 | 156.1 | 149.0 | 163.2 |
| min: 14 / without RS | 0.13 | -0.06 | 0.33 | 72.3 | 69.7 | 74.9 | 95.1 | 93.3 | 96.8 | 150.0 | 144.7 | 155.3 |
| min: 14 / with RS | 0.04 | -0.28 | 0.36 | 73.8 | 70.5 | 77.2 | 91.9 | 89.7 | 94.0 | 155.5 | 148.4 | 162.6 |
| min: 15 / without RS | 0.00 | -0.20 | 0.20 | 71.3 | 68.7 | 73.9 | 95.5 | 93.8 | 97.2 | 150.3 | 145.0 | 155.6 |
| min: 15 / with RS | 0.00 | -0.32 | 0.32 | 74.0 | 70.6 | 77.3 | 92.5 | 90.3 | 94.6 | 156.9 | 149.8 | 164.0 |
| time*GA |  |  |  |  |  |  |  |  |  |  |  |  |
| min: 2 / term |  |  |  | 56.1 | 53.7 | 58.6 | 67.8 | 66.1 | 69.5 | 116.3 | 110.8 | 121.9 |
| min: 2 / preterm |  |  |  | 53.9 | 50.7 | 57.1 | 67.1 | 65.0 | 69.3 | 109.7 | 103.0 | 116.4 |
| min: 3 / term |  |  |  | 55.4 | 53.0 | 57.8 | 70.3 | 68.7 | 71.8 | 130.6 | 125.6 | 135.5 |
| min: 3 / preterm |  |  |  | 53.2 | 50.0 | 56.3 | 69.6 | 67.5 | 71.6 | 124.0 | 117.7 | 130.2 |
| min: 4 / term |  |  |  | 58.5 | 56.2 | 60.9 | 73.3 | 71.8 | 74.8 | 145.7 | 140.9 | 150.5 |
| min: 4 / preterm |  |  |  | 56.3 | 53.2 | 59.4 | 72.6 | 70.6 | 74.6 | 139.1 | 133.0 | 145.2 |
| min: 5 / term |  |  |  | 62.2 | 59.9 | 64.6 | 77.8 | 76.2 | 79.3 | 150.4 | 145.6 | 155.2 |
| min: 5 / preterm |  |  |  | 60.0 | 56.9 | 63.1 | 77.1 | 75.0 | 79.1 | 143.8 | 137.7 | 149.8 |
| min: 6 / term |  |  |  | 65.9 | 63.6 | 68.2 | 82.0 | 80.5 | 83.6 | 151.6 | 146.9 | 156.4 |
| min: 6 / preterm |  |  |  | 63.6 | 60.5 | 66.7 | 81.3 | 79.3 | 83.4 | 145.0 | 139.0 | 151.1 |
| min: 7 / term |  |  |  | 68.6 | 66.3 | 70.9 | 85.8 | 84.3 | 87.4 | 151.9 | 147.1 | 156.7 |
| min: 7 / preterm |  |  |  | 66.3 | 63.3 | 69.4 | 85.1 | 83.1 | 87.2 | 145.3 | 139.2 | 151.4 |
| min: 8 / term |  |  |  | 71.1 | 68.8 | 73.4 | 88.9 | 87.4 | 90.4 | 153.6 | 148.8 | 158.4 |
| min: 8 / preterm |  |  |  | 68.8 | 65.7 | 71.9 | 88.2 | 86.2 | 90.3 | 147.0 | 140.9 | 153.0 |
| min: 9 / term |  |  |  | 71.8 | 69.4 | 74.1 | 90.3 | 88.8 | 91.8 | 153.2 | 148.4 | 157.9 |
| min: 9 / preterm |  |  |  | 69.5 | 66.4 | 72.6 | 89.6 | 87.6 | 91.7 | 146.6 | 140.5 | 152.6 |
| min: 10 / term |  |  |  | 72.7 | 70.4 | 75.1 | 91.5 | 90.0 | 93.0 | 155.0 | 150.2 | 159.8 |
| min: 10 / preterm |  |  |  | 70.5 | 67.4 | 73.6 | 90.8 | 88.8 | 92.9 | 148.4 | 142.3 | 154.5 |
| min: 11 / term |  |  |  | 73.5 | 71.1 | 75.8 | 92.4 | 90.8 | 93.9 | 154.8 | 150.0 | 159.6 |
| min: 11 / preterm |  |  |  | 71.2 | 68.1 | 74.3 | 91.7 | 89.6 | 93.7 | 148.2 | 142.1 | 154.3 |
| min: 12 / term |  |  |  | 74.2 | 71.9 | 76.5 | 93.0 | 91.5 | 94.6 | 156.1 | 151.3 | 160.9 |
| min: 12 / preterm |  |  |  | 71.9 | 68.9 | 75.0 | 92.3 | 90.3 | 94.4 | 149.5 | 143.4 | 155.6 |
| min: 13 / term |  |  |  | 74.6 | 72.3 | 76.9 | 93.7 | 92.2 | 95.2 | 155.9 | 151.1 | 160.7 |
| min: 13 / preterm |  |  |  | 72.3 | 69.3 | 75.4 | 93.0 | 91.0 | 95.1 | 149.3 | 143.2 | 155.4 |
| min: 14 / term |  |  |  | 74.2 | 71.9 | 76.5 | 93.8 | 92.3 | 95.3 | 156.0 | 151.2 | 160.8 |
| min: 14 / preterm |  |  |  | 71.9 | 68.8 | 75.0 | 93.1 | 91.1 | 95.1 | 149.4 | 143.3 | 155.5 |
| min: 15 / term |  |  |  | 73.7 | 71.4 | 76.1 | 94.3 | 92.8 | 95.8 | 156.9 | 152.1 | 161.7 |
| min: 15 / preterm |  |  |  | 71.5 | 68.4 | 74.6 | 93.6 | 91.6 | 95.7 | 150.3 | 144.2 | 156.4 |

*RS*, respiratory support*; GA*, gestational age; *95%CI*, 95% confidence intervals; *CBV*, cerebral blood volume; *cTOI*, cerebral tissue oxygenation index; *HR*, heart rate; *SpO_2_*, arterial oxygen saturation
